# Supplementary material for: Biomarker-Based Responder Selection and Early Prediction of Treatment Response in Hepatocellular Carcinoma: Dynamic Changes in Alpha-Fetoprotein and Des-Gamma-Carboxy Prothrombin During Atezolizumab Plus Bevacizumab Therapy
Source: Cancers (Basel). 2025 Dec 5;17(24):3891. doi: 10.3390/cancers17243891 (PMC12730933; doi:10.3390/cancers17243891)
Supplement: Supplementary file 1 [file cancers-17-03891-s001.zip › cancers-3978407-supplementary.pdf]

Table S1. Predictive performance of tumor marker ratios at week 3 for 1st-PD

Marker	AUC	Cutoff	Sensitivity	Specificity	NPV	LR−
AFP ratio	0.87	≥1.4*	0.93	0.81	0.97	0.10
DCP ratio	0.70	≥1.0*	0.67	0.72	0.85	0.46
AFP ≥1.4 or DCP ≥1.0 (combined)	–	–	0.93	0.63	0.97	0.11
AFP ≥1.4 and DCP ≥1.0 (combined)	–	–	0.67	0.90	0.88	0.37

Abbreviations: AUC, area under the curve; AFP, alpha-fetoprotein; DCP, des-γ-carboxy prothrombin; NPV, negative predictive value; LR−, negative likelihood ratio; PD, progressive disease.

Notes: Optimal cutoffs by ROC analysis were 1.41 for AFP and 0.92 for DCP. For clinical applicability, these were rounded to AFP ≥1.4 and DCP ≥1.0 to provide practical thresholds and to avoid sub-baseline values being misclassified as progression. Values represent performance for predicting progressive disease (PD) at first evaluation (week 6) based on tumor marker ratios at 3 weeks.

Table S2. Predictive performance of tumor marker ratios at week 9 for 2nd-PD

Marker	AUC	Cutoff	Sensitivity	Specificity	NPV	LR−
AFP ratio	0.78	≥1.1*	0.63	0.84	0.88	0.45
DCP ratio	0.75	≥1.5*	0.75	0.74	0.93	0.34
AFP ≥1.1 or DCP ≥1.5 (combined)	–	–	0.93	0.68	0.97	0.10
AFP ≥1.1 and DCP ≥1.5 (combined)	–	–	0.50	0.90	0.87	0.56

Abbreviations: AUC, area under the curve; AFP, alpha-fetoprotein; DCP, des-γ-carboxy prothrombin; NPV, negative predictive value; LR−, negative likelihood ratio; PD, progressive disease.

Notes: Optimal cutoffs by ROC analysis were AFP 1.11 and DCP 1.50. For clinical applicability, these were rounded to AFP ≥1.1 and DCP ≥1.5 to provide practical thresholds and to avoid sub-baseline values being misclassified as progression. Values represent performance for predicting progressive disease (PD) at the second evaluation (median 14.8 weeks) based on tumor marker ratios at 9 weeks.
